# Screening and Action Mechanism of Biological Control Strain *Bacillus atrophaeus* F4 Against Maize Anthracnose

**DOI:** 10.3390/microorganisms14010047

**Published:** 2025-12-25

**Authors:** Pengfei Wang, Yingying Xi, Ke Liu, Jiaqi Wang, Qiubin Huang, Haodong Wang, Shaowei Wang, Gang Wang, Nuerguli Reheman, Fengying Liu

**Affiliations:** 1 Xinjiang Key Laboratory of Special Species Conservation and Regulatory Biology, School of Life Sciences, Xinjiang Normal University, Urumqi 830054, China; 2 Henan Key Laboratory of Synthetic Biology and Biomanufacturing, Henan University, Kaifeng 475004, China; 3 Joint National Laboratory for Antibody Drug Engineering, Henan University, Kaifeng 475004, China; 4 School of Life Sciences, Henan University, Kaifeng 475004, China; 5 Engineering Research Center for Applied Microbiology of Henan Province, Kaifeng 475004, China

**Keywords:** *Bacillus atrophaeus*, biocontrol, antagonisms, lipopeptide extract, *Colletotrichum graminicola*

## Abstract

Anthracnose caused by *Colletotrichum graminicola* (Ces.) G.W.Wils is a significant disease of maize (*Zea mays*) worldwide. To obtain an efficient biocontrol strain and elucidate its mechanisms, 103 bacterial isolates were obtained from soil samples collected in the Tianshan Mountains, Xinjiang, China. Among these, *Bacillus atrophaeus* F4’s fermentation broth had the highest efficacy in controlling maize anthracnose, reaching 79.78%. To further investigate biocontrol mechanisms of F4 strain, its complete genome was sequenced, assembled, and annotated. Lipopeptides extracted from the fermentation broth of F4 were found to strongly inhibit the growth of hyphae and the germination of conidia in the pathogen. Microscopic and biochemical analyses indicated that the lipopeptide extract inhibited chitin synthesis and disrupted the integrity of the cell wall and membrane, thereby exerting antifungal effects. Further MALDI-TOF MS analysis identified antimicrobial compounds, including surfactin, iturin, and fengycin B, in the lipopeptide extract. Furthermore, plate antagonistic test showed that F4 strain exhibited broad-spectrum antagonistic activity against multiple plant pathogenic fungi. F4 strain also displayed motility, biofilm-forming capacity, and the ability to produce extracellular enzymes such as proteases and amylases, which are associated with biocontrol activity. These findings suggest the significant potential of *B. atrophaeus* F4 as a biocontrol agent against maize anthracnose.

## 1. Introduction

Maize (*Zea mays* L.), a globally vital staple and forage crop, with a global production exceeding 11.6 billion tons annually [1], plays a pivotal role in ensuring food security. However, its cultivation is severely threatened by anthracnose, a disease caused by the fungal pathogen *Colletotrichum graminicola* [2,3,4]. The disease was first reported in Italy in 1852 [5] and has since spread globally. Recently, Switzerland [6], China [7], Portugal [8], Galicia (northwestern Spain) [9], and Austria [10] have reported the occurrence of this disease, indicating that its geographical scope is expanding and may further exacerbate global food security risks [11]. Anthracnose causes significant yield losses in maize, primarily manifested as anthracnose leaf blight (ALB) and anthracnose stalk rot (ASR). ALB reduces photosynthetic area, while ASR leads to plant lodging. In the United States and Canada, annual yield losses due to ASR amount to 4.08 million metric tons (approximately 1% of total maize production), while ALB results in a reduction of about 0.13 million metric tons (approximately 0.03% of total production) [12].

In recent years, significant advancements have been achieved in the fundamental biological research of this pathogen. Genomic analyses have revealed substantial genetic diversity and complex population structures in *C. graminicola*, as well as its phylogenetic relationships with other *Colletotrichum* species that cause anthracnose in various hosts [11,13,14]. Specifically, recent population genomics studies indicate that *C. graminicola* exhibits clear genetic differentiation across geographical regions, while also showing evidence of long-distance migration and local adaptation [11]. Earlier phylogenetic work further supports the distinct evolutionary trajectory of maize-infecting lineages within the genus [13], and newly sequenced field strains continue to enrich our understanding of its genomic plasticity and pathogenic potential [14]. Key virulence factors, including Cgf1 [15], NPS6 [16], and Snf1 [17], have been identified, gradually elucidating infection mechanisms. The application of Agrobacterium mediated transformation (ATMT) and CRISPR/Cas9 gene editing technology provides effective tools for in-depth analysis of gene function and pathogenic mechanisms [18,19].

Nevertheless, effective control of maize anthracnose remains a significant challenge. Current management strategies predominantly involve five principal approaches: (i) Chemical control, which has historically been the primary method, is associated with significant risks, including the development of pathogen resistance, pesticide residues, environmental damage, and toxicity to non-target organisms such as beneficial microbes and pollinators. (ii) Cultivation of resistant cultivars. The resistance of maize to anthracnose includes quality resistance controlled by a single gene and quantity resistance controlled by multiple genes. Several resistance genetic sources have been identified, such as LB1, LB6 resistant to ALB, and A556, MP305 resistant to ASR [20]. However, long-term use of a single disease resistant variety will exert targeted selection pressure, leading to the dominance of toxic small breeds and triggering disease outbreaks. In addition, the intercontinental migration of pathogens may introduce new toxic genes, exacerbating the difficulty of resistance breedinr. (iii) Cultural practices. Maize residues are important carriers for the survival and initial invasion of *C. graminicola* during different seasons [21]. No-till and continuous cropping can increase the pathogenic fungi in the field, whereas tillage and rotation reduce inoculants and alleviate the occurrence of diseases [22,23]. (iv) Plant defense. Studies indicate that broad-spectrum defense hormones including jasmonic acid (JA) and salicylic acid (SA) can regulate maize defense against *C. graminicola*. Maize-derived green leaf volatiles (GLVs) can induce JA biosynthesis, thereby enhancing JA signaling-mediated defenses. However, due to the strong antagonism between JA and SA signaling, this GLVs-JA positive feedback loop may suppress SA-dependent defenses, potentially increasing maize susceptibility to anthracnose [24,25]. (v) Biological control. It represents an environmentally friendly and sustainable strategy to replace or complement chemical fungicides. Although microbial resources have garnered significant interest, the exploration of strains with potential against maize anthracnose remains limited. Studies have reported that *Trichoderma virens* secretes the proteinaceous elicitor Sm1 during maize root colonization, which acts as a key signaling molecule to trigger induced systemic resistance (ISR) against the pathogen *C. graminicola*. Sm1 is a hydrophobin-like protein that does not exhibit direct antimicrobial activity but functions as a non-enzymatic elicitor of plant defense responses [26]. Similarly, the rhizobacterium *Pseudomonas putida* KT2440 acts as an inducer, may establish a defense activation state by activating the jasmonic acid/ethylene dependent defense pathway, leading to a comparable defensive response upon root colonization [27]. Furthermore, earlier research has indicated the potential of *Saccharomyces cerevisiae* and phyllosphere yeasts in protecting maize from this pathogen [28,29].

Currently, the biological control of maize anthracnose faces challenges such as limited biocontrol resources, inconsistent efficacy, and unclear mechanisms. Thus, screening highly efficient and stable biocontrol strains and elucidating their mechanisms are essential prerequisites for practical application. Based on the above background, we hypothesize that bacterial strains isolated from the Tianshan Mountain soils possess potent antagonistic activity against *C. graminicola*, primarily through the production of antifungal lipopeptides that disrupt fungal cell walls and membranes, thereby suppressing hyphal growth and conidial germination. The in-depth study of this mechanism of action provides a theoretical basis and microbial resources for the development of green control strategies for this disease.

## 2. Materials and Methods

### 2.1. Plant Material and Strain Culture

The susceptible Zea mays line B73 was used in this study.

Bacterial strains and culture. The bacterial strain F4, which was the primary focus of subsequent experiments, was isolated and screened from soil samples collected in the Tianshan Mountains, Xinjiang, China as described in Section 2.2 and Section 2.3. The other bacterial isolates in this study were also obtained from the same soil sampling and isolation procedure. Bacterial strains were routinely cultured in lysogeny broth (LB, 10 g tryptone, 5 g yeast extract, and 10 g NaCl per liter, pH 7.0) solid or liquid medium at 30 °C.

For the preparation of fermentation broth, bacterial strains were first activated on LB agar plates. A single colony was then inoculated into LB liquid medium and incubated under the conditions described above for 16–24 h. The resulting culture, containing both cells and extracellular metabolites, is referred to as “bacterial fermentation broth” and was used directly in greenhouse screenings.

When bacterial suspensions of F4 strain were required, log-phase cells were collected, washed with sterile phosphate-buffered saline (PBS, pH 7.4), and resuspended in PBS to an optical density at 600 nm (OD_600_) of 0.5.

Fungal strains. Eight plant-pathogenic fungi were used, including *C. graminicola*. All fungal strains were obtained from and preserved in our laboratory culture collection. Their full names, standard abbreviations, and the major diseases they cause (with supporting references) are listed in Table 1. All fungi were cultured on potato dextrose agar (PDA) medium at 25 °C for 4–10 days. PDA was prepared by boiling 200 g of potato extract and adding 20 g of dextrose and 15 g of agar per liter of distilled water.

To obtain conidia of *C. graminicola*, the well-grown fungal mycelia on PDA plate was inoculated into liquid complete medium containing 0.5 M sucrose and shaken at 80 rpm, 23 °C for 2 days, followed by incubation in darkness for 5 days to produce conidia [37]. The cultures were filtered through four layers of sterile gauze to remove mycelia, and the resulting conidia were washed with PBS and resuspended in PBS to a final concentration of 1 × 10^5^ conidia/mL.

### 2.2. Sample Collection and Bacterial Strain Isolation

Sampling method and soil characteristics. Rhizosphere soil samples were collected from an uncultivated natural grassland at the foothills of the Tianshan Mountains in Xinjiang, China (41°15′36″ N, 80°12′45″ E) on 1 March 2024. Soil was collected from a depth of 5–20 cm using a sterile spatula from five randomly selected points within a 10 m × 10 m area. Subsamples were composited in a sterile bag. The site has no history of agricultural cultivation, ensuring the sampled microbial community was free from direct anthropogenic input. The soil was characterized as a sandy loam mountain meadow soil (Cambisol) with a pH of 7.2 (measured in 1:2.5 soil–water suspension). Organic matter content was visually assessed as moderate to high, consistent with the undisturbed grassland vegetation. Immediately after collection, the samples were placed in sterile plastic bags and stored on ice to maintain stability.

Bacterial Strain Isolation. A total of 5 g of soil was suspended in 45 mL of sterile water. After serial dilution to an appropriate concentration, 100 μL of the suspension was spread onto LB agar plates and incubated at 30 °C for 24–48 h. The bacterial colonies that appeared were purified by streaking on LB agar plates. Pure isolates were subsequently inoculated into 5 mL LB broth and cultured at 30 °C, 180 rpm for 16 h. The bacterial cultures were then mixed with sterile glycerol to a final concentration of 30% (*v*/*v*) and stored at −80 °C for subsequent experiments.

### 2.3. Greenhouse Screening of Biocontrol Bacteria

The biocontrol efficacy of bacterial strains against maize anthracnose was evaluated on seedlings. Maize seeds were surface-sterilized with 75% ethanol, germinated, and sown in plastic pots containing sterile soil. Four weeks later, transfer the seedlings to a greenhouse (humidity: 80% ± 10%; photoperiod: 12/12 h; temperature: 26/18 ± 2 °C) for biocontrol testing. Bacterial and pathogen application followed a sequential schedule:

Day 1: Seedlings were treated by spraying 30 mL of stationary-phase bacterial fermentation broth onto the leaves.

Day 2: Plants were challenged by spraying 30 mL of a *C. graminicola* conidial suspension (1 × 10^5^ conidia/mL) onto the leaves.

Day 3: A second application of the bacterial fermentation broth (30 mL) was performed.

Only conidia suspension and sterile LB medium were utilized as positive and negative controls, respectively. After one week of inoculation, disease severity was investigated according to a 5-level grading standard, and the biocontrol efficacy of bacteria were calculated [38]. The bacterial strain exhibiting the highest and consistent biocontrol efficacy was selected as the target biocontrol strain for further molecular and genomic analyses. The remaining isolates, which showed lower or inconsistent activity, were archived.

### 2.4. Strain Identification and Genome Sequencing

Cultivate the bacteria with LB broth until the log phase, collect the bacterial cells, and extract the whole genome DNA. Strain identification was performed by 16S rDNA sequencing (Sangon Biotech, Shanghai, China). Briefly, the 16S rRNA gene fragment was amplified using universal primers fD1 (5′-AGAGTTTGATCCTGGCTCAG-3′) and rP2 (5′-TACGGCTACCTTGTTACGACTT-3′) [39]. The PCR products were purified, recovered, and subsequently sequenced. The resulting sequence was aligned by BLAST (https://blast.ncbi.nlm.nih.gov/Blast.cgi, accessed on 21 December 2025) in NCBI database. A phylogenetic tree was constructed employing MEGA 11 software with the neighbor-joining method and a bootstrap value of 1000 [40].

Whole-genome sequencing and analysis were completed by Frasergen Bioinformatics Co., Ltd. (Wuhan, China). Sequencing was performed on a PacBio Sequel II platform (Pacific Biosciences, Menlo Park, CA, USA), with subsequent data processing and conversion into HiFi read samples using SMRT Link 11.0.0 software, followed by assembly with Flye-2.9 software [41]. Functional annotation was executed using the NR, COG/KOG, GO, SwissProt, KEGG, and InterPro databases. Prediction of secondary metabolite gene clusters was accomplished using antiSMASH 7.0 [42].

### 2.5. Biocontrol Experiment on Detached Maize Leaves

The inoculation assays on detached maize leaves were performed as described previously, with minor modifications [37]. Specifically, the second leaves from 16-day-old maize plants were excised [43] and placed on moist filter paper. A mixture of 10 μL conidial suspension and 10 μL lipopeptide extract from bacterial fermentation supernatant was spotted at 3–4 points along the midvein of the leaf surface. Leaves treated with only the conidial suspension or sterile water served as positive and negative controls, respectively. The leaves were maintained in a humid chamber at 25 °C (>90% relative humidity) with a 12 h photoperiod. Disease symptoms were evaluated after 5 days of incubation.

### 2.6. Conidial Germination Inhibition Assay

The inhibitory effects of the lipopeptide extract on conidial germination were evaluated based on the method reported [44], with modifications. The specific procedures are as follows:

Agar well diffusion assay: A conidial suspension (1 mL) was mixed with PDA medium pre-melted at 40 °C and poured into Petri dishes. After solidification, Oxford cups (Ertraee, Beijing, China) were placed on the agar surface and filled with 50 μL of the 100 μg/mL lipopeptide extract, while sterile water was used as a negative control. The plates were incubated at 28 °C for 24 h, and the formation of clear inhibition zones around the Oxford cups indicated conidial germination inhibition.

Dynamic observation of conidial germination: A conidial suspension (20 μL) was mixed with 20 μL of the 100 μg/mL lipopeptide extract on a glass slide, with an equivalent volume of sterile water-treated conidial suspension serving as control. The slides were incubated at 28 °C, and conidial germination progress was monitored and recorded every 6 h under Leica DM4 B microscopy (Leica, Düsseldorf, Germany).

### 2.7. Extraction of Lipopeptides and MALDI-TOF MS Analysis

According to previous method reported [45,46], lipopeptides were extracted from the supernatant of cell fermentation broth and analyzed by MALDI-TOF MS. Specifically, the activated F4 strain was inoculated into 1000 mL of LB liquid medium at a 1:100 (*v*/*v*) ratio and cultured at 30 °C, 200 rpm for 24 h. The cells were removed by centrifugation (5000× *g*, 20 min, 4 °C), and the supernatant was then acidified to pH 2.0 using concentrated HCl and kept at 4 °C overnight to precipitate lipopeptides. After centrifugation under the same conditions, the obtained precipitate thoroughly suspended in methanol with continuous stirring of 2 h for extraction. The resulting extract was filtered through a 0.45 μm organic membrane and concentrated to complete dryness using a rotary evaporator (Great Wall Scientific, Zhengzhou, China). The resulting solid particles were redissolved in methanol for subsequent MALDI-TOF MS analysis. Additionally, part of the extract was diluted with sterile water to prepare a crude lipopeptide extract solution for antibacterial activity analysis. All cultivation and extraction procedures were independently performed in duplicate.

MALDI-TOF MS analysis of lipopeptide was made on a Bruker Daltonics MALDI-ToF-MS Ultraflex (Bruker Daltonics, Bremen, Germany) under positive reflection mode. Samples are ionized with a 50 Hz nitrogen laser (λ = 337 nm), accelerating voltage 20 kV. The mass spectra were recorded in the range of 1000–1500 *m*/*z* with 1000 laser shots. Finally, the mass of molecular ion adducts peak were calculated by MassLynx software (4.1 version, Waters, Milford, MA, USA) and compared with previously published literature.

### 2.8. Microscopy Techniques

For scanning electron microscopy (SEM) analysis, the morphology of bacterial strain F4 was examined following a previously established protocol [47]. The morphology of fungal hyphae was assessed using a modified version of an existing method [48]. Specifically, approximately 5 mm × 5 mm fungal blocks were excised from the edge of PDA plate colonies and immediately immersed in pre-cooled 0.05 M PBS (pH 6.8) containing 2.5% glutaraldehyde for 6 h fixation at 4 °C. The samples were then rinsed three times with PBS buffer to remove residual fixative, followed by gradient ethanol dehydration and critical point drying with CO_2_. Finally, the hyphae were coated with gold particles with an ion sputter coater (Quorum Technologies, Lewes, UK) and observation with a JEOL JSM-7610F Plus scanning electron microscope (Hitachi, Tokyo, Japan).

For fluorescence microscopy, fungal hyphae were stained with Calcofluor White (CFW, Sigma F3543, Sigma Aldrich, St. Louis, MO, USA) to evaluate cell wall structure. Specifically, 20 µM CFW directly applied to hyphae grown on PDA plates and stained for 20 min at 28 °C under dark conditions. Then washed twice with PBS buffer. The blue fluorescence intensity of the hyphae was then examined using fluorescence microscopy [49]. The membrane integrity of fungal conidia was assessed using propidium iodide (PI, Cat. no: P1304MP) staining. In brief, the fungal conidia were washed twice with PBS buffer, resuspended in a PBS solution containing 10 µg/mL PI, and incubated at 28 °C in the dark for 20 min. Following incubation, the conidia were again washed twice with PBS buffer, and the red fluorescence intensity was observed using a Axio Imager.A2 fluorescence microscope (Zeiss, Jena, Germany) [50].

### 2.9. Determination of Chitin Content in Hyphal Cell Walls

Quantitative analysis of chitin in mycelium refers to reported method [44]. The fungal was inoculated in potato dextrose broth (PDB) liquid medium and incubated at 25 °C with shaking for 4 days. Mycelia were subsequently harvested by filtration through four layers of sterile gauze. The experimental group was treated with 2 mL of lipopeptide extract for 24 h, while the control group used an equal volume of sterile water. After treatment, the mycelia were dried at 80 °C for 3 h and then ground into fine powder, with the initial weight recorded as W1. The samples were then subjected to an alkaline treatment using a saturated potassium hydroxide (KOH) solution at 140 °C for 60 min to eliminate non-chitin components. This was followed by repeated washing with distilled water until the pH reached neutrality (pH 7.0). The precipitate was collected via centrifugation and subsequently dehydrated using 95% and 100% ethanol. The final dried residue was weighed and recorded as W2. The chitin content was calculated using the formula: Chitin content (%) = (W2/W1) × 1.26 × 100%, where 1.26 serves as the conversion factor.

### 2.10. Plate Confrontation Assay

To evaluate the antagonistic activity of F4 strain against phytopathogenic fungi, the plate confrontation method was employed [51]. Fungal disks (8 mm diameter) were punched at the edges of activated fungi and then placed at the center of a freshly prepared PDA plate. In total, 5 μL bacteria suspension (OD_600_ = 0.5) was inoculated 2.5 cm away from the fungal disks. Only fungal inoculation was used as control. The plates were cultured at 25 °C for 4–10 days. The diameters of the treatment colonies were measured, and the inhibition rate was calculated using the following formula: Inhibition rate (%) = [(control diameter − treatment diameter)/(control diameter − 0.08)] × 100%.

### 2.11. Motility, Biofilm, and Extracellular Enzymes Assays

The motility assay was performed according to a previously described method. A total of 5 µL of bacterial suspension transferred onto semi-solid agar plates and incubated statically at 30 °C for 6 h. The motility halo surrounding the bacterial strain was observed, and the surrounding bacteria were collected for silver nitrate staining to assess the presence of flagella.

Biofilm formation was assessed according to the established crystal violet staining protocol of [52] with a modification in the final quantification step. In our study, the crystal violet-destaining solution (ethanol–propanone = 7:3, *v*/*v*) was subjected to a 10-fold dilution to ensure that the absorbance readings at 570 nm fell within the optimal linear range of the spectrophotometer.

For the extracellular protease assay, 5 µL of bacterial suspension was inoculated onto LB plates supplemented with 1% skim milk powder and incubated at 30 °C for 2 days. The presence of hydrolytic transparent zone around the colony indicates that the bacteria have the ability to produce proteases.

The extracellular amylase assay was performed according to a previously described method [53].

### 2.12. Statistical Analysis

The data obtained were statistically analyzed using GraphPad Prism 8.0. Student’s *t*-test or Analysis of variance (ANOVA) was used to verify significant differences between groups. All experiments were conducted in triplicate, and the results are presented as mean ± SD (*n* = 3). Different lowercase letters represent significant differences. Significance is indicated on figures as follows: ** *p* < 0.01; *** *p* < 0.001.

## 3. Results

### 3.1. In Vivo Screening and Identification of Biocontrol Strains Against Maize Anthracnose

**Figure 1:**
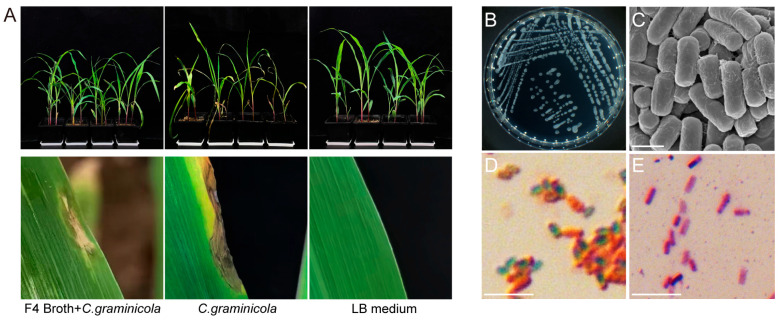
Biocontrol potential and morphological characterization of *B. atrophaeus.* F4. (**A**) F4 strain against maize anthracnose. The disease symptoms in the F4-treated plants were significantly reduced compared to the positive control (inoculated only with *C. graminicola*) within glasshouse conditions. Sterile LB medium treatment was used as negative control, and F4 Broth denotes F4 fermentation broth. (**B**) Colony morphology on an LB agar plate. (**C**) Scanning electron microscopy (SEM) image of bacterial cells. Scale bar, 1 µm. (**D**) Endospore staining. Vegetative cells appear red, while endospores appear green. Scale bar, 10 µm. (**E**) Gram staining shows a positive (purple) reaction. Scale bar, 10 µm.

A total of 103 bacterial isolates with distinct morphological characteristics were obtained from soil samples. The in vivo screen identified that 12 isolates significantly reduced disease incidence and severity, 9 isolates exacerbated disease, and 82 isolates showed no significant effect compared to the positive control. Among the effective isolates, F4 strain demonstrated the most significant control effect (Figure 1A), achieving a 78.95% reduction in disease incidence and a 79.78% control efficacy (Table 2). Considering the promising biocontrol performance of F4 strain, it was selected as a biocontrol strain for subsequent research.

**Figure 2:**
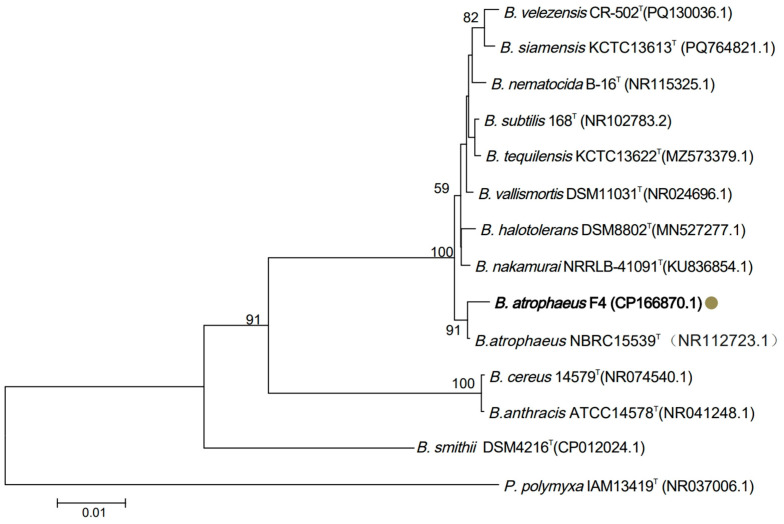
Phylogenetic analysis of F4 strain based on 16S rRNA gene sequence. The phylogenetic tree was constructed using the neighbor-joining method with 1000 bootstrap replicates in MEGA 11.0 software. F4 Strain is highlighted with a solid circle. The scale bar indicates the number of nucleotide substitutions per site. Sequences of type strains were obtained from the NCBI GenBank database.

F4 strain formed white, dry, and rough circular colonies on LB agar plates, with wrinkled surfaces, turning dark brown after 20 days. Gram staining was positive, and sporulation was observed. Scanning electron microscopy revealed short rod-shaped cells approximately 1–2 μm in length (Figure 1B–E). Full-length 16S rRNA gene sequencing analysis indicated that F4 strain shared 91% sequence similarity with *B. atrophaeus* 15539. Phylogenetic tree analysis further clustered F4 strain within the *B. atrophaeus* clade (Figure 2). Based on these morphological and genetic characteristics, F4 strain was conclusively identified as *B. atrophaeus.*

### 3.2. Complete Genome Sequencing and Analysis of B. atrophaeus F4

The whole-genome sequencing data have been deposited in the NCBI database under the accession number CP166870.1. Genome sequencing was performed on a PacBio Sequel II platform, generating 30,402 high-fidelity (HiFi) reads with an N50 length of 10,632 bp, providing an average coverage depth of 77.13×.

**Figure 3:**
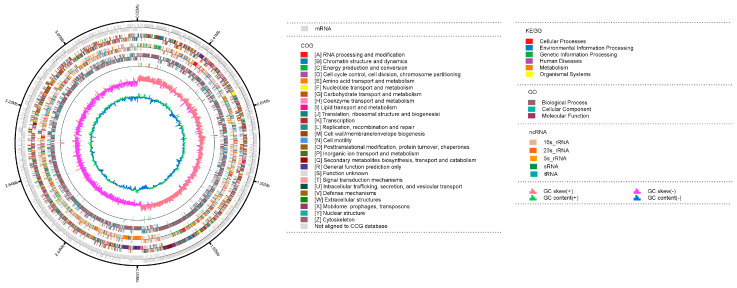
Circular genome visualization map of the *B. atrophaeus* F4. Genome generated using the Circos server (version 0.69-9). From outside to the center, rings 1–2: Protein-coding genes on forward and reverse strands, respectively. Rings 3–4, rings 5–6, rings 7–8, and rings 9–10 show genes colored by functional categories (COG, KEGG, GO) and ncRNA categories, respectively. Ring 11: GC skews. Ring 12: G + C% content plot (blue indicates values above the genomic average, and green indicates values below the average).

The F4 genome consists of a single circular chromosome (Figure 3) with a total length of 4,059,320 bp, exhibiting an average GC content of 43.5%. The assembly exhibited high continuity, with an N50 (and L50) value of 4,059,320 bp (representing the single contig). Assessment of assembly quality based on HiFi read mapping showed 100% coverage of the genome sequence.

The genome was predicted to contain 4209 genes, including 3830 protein-coding sequences (CDSs), 82 tRNA genes, and 24 rRNA genes (8 copies each of 5S, 16S, and 23S rRNA). Functional annotation assigned 96.06% (4043) of the predicted genes to the Non-Redundant (NR) protein database. Among these, 99.38% shared highest similarity with genes from *B. atrophaeus*, confirming its taxonomic placement. This was further supported by Average Nucleotide Identity (ANI) analysis, which showed that strain F4 shares 99.11% ANI with the *B. atrophaeus* type strain NBRC 15539^T^ (accession number NR112723.1), unequivocally identifying it at the species level.

Comprehensive functional profiling was performed. Cluster of Orthologous Groups (COG) annotation classified 77.29% of the genes into 25 functional categories, with the majority involved in Metabolism, Transcription, and Cellular processes and signaling. KEGG pathway analysis indicated enrichment in metabolic pathways related to carbon metabolism, biosynthesis of amino acids, and secondary metabolites. Gene Ontology (GO) terms were assigned to 76.36% of the genes, spanning biological processes, molecular functions, and cellular components.

AntiSMASH analysis of *B. atrophaeus* F4 genome predicted 11 putative biosynthetic gene clusters (BGCs) associated with secondary metabolism (Table 3). Among these, six BGCs demonstrated high similarity to known clusters involved in the synthesis of specific metabolites. Specifically, five BGCs were identified as nonribosomal peptide synthetases (NRPS), and one as a sactipeptide. These clusters were linked to the production of specialized metabolites, including sporulation killing factor, surfactin, bacillaene, fengycin, and subtilosin A. The seventh cluster showed low similarity to the BGC associated with the production of 1-carbapen-2-em-3-carboxylic acid. The remaining four clusters, classified as terpene, azole-containing ribosomally synthesized and post-translationally modified peptide (RIPP), and epipeptide, exhibited no homology to any known gene clusters, suggesting the potential for novel secondary metabolite production in F4 strain.

### 3.3. F4 Lipopeptide Extract Exhibits Biocontrol Activity Against Maize Anthracnose in Detached Leaves

To investigate the bioactive compounds responsible for the biocontrol activity of F4 strain, we first evaluated the efficacy of its cell-free fermentation supernatant against maize seedling anthracnose under greenhouse conditions. The results demonstrated that the supernatant significantly reduced the disease index and incidence rate, achieving a control efficacy of 77.53% (Table 2). Furthermore, Plate antagonism assays confirmed that the supernatant inhibited the mycelial growth of *C. graminicola*. Genomic analysis indicated that F4 strain harbors three biosynthetic gene clusters responsible for lipopeptide antimicrobial metabolites, implying that its biocontrol activity may be associated with lipopeptide compounds.

**Figure 4:**
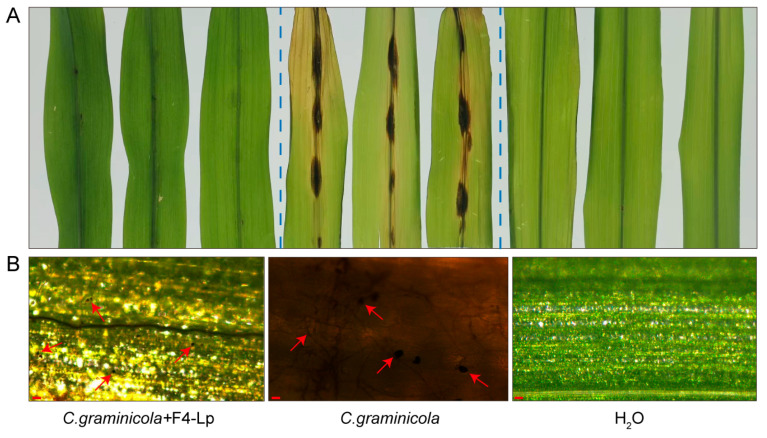
Biocontrol activity of lipopeptide extract from *B. atrophaeus* F4 against maize anthracnose on detached leaves. The treatment group was co-inoculated with *C. graminicola* conidia and the lipopeptide extract. The positive control was inoculated with pathogen conidia alone, and the negative control was treated with sterile water. Labels: F4-Lp, F4 lipopeptide extract. (**A**) Disease symptoms on maize leaves. (**B**) Microscopic observation of leaf cross-sections. The red arrow indicates *C. graminicola* conidia. Scale bar, 20 µm.

To validate this hypothesis, lipopeptides were extracted and their biocontrol efficacy was assessed on detached leaves. In the positive control group, which was inoculated only with pathogen conidia, the inoculation sites exhibited distinct water-soaked lesions that expanded longitudinally along the midvein. After five days, these lesions developed into dark brown necrotic spots, some of which coalesced, displaying typical symptoms of maize anthracnose. In contrast, treatment with the F4 lipopeptide extract markedly alleviated these symptoms. The negative control, treated with sterile water, showed no lesions, with only slight water-soaked traces at the inoculation sites and no signs of chlorosis or necrosis (Figure 4A). Quantitative assessment revealed that the average lesion length in the positive control group reached 13.0 ± 1.7 mm, whereas in the F4 lipopeptide-treated group, it was significantly reduced to 1.6 ± 0.5 mm (*p* < 0.01). Additionally, disease incidence was 96% in the positive control, compared to only 15% in the lipopeptide-treated group.

Microscopic examination of leaf cross-sections revealed that the positive control lesions contained black conidia, accompanied by extensive hyphal proliferation (varying from colorless to dark brown) within the leaf tissues, resulting in the formation of necrotic foci. In contrast, the group treated with the F4 lipopeptide extract exhibited only black conidia, with no signs of conidial germination, hyphal invasion, or tissue necrosis. The negative control maintained intact leaf tissue structures without any pathogen colonization (Figure 4B).

In summary, the lipopeptide components in the F4 fermentation supernatant demonstrated strong biocontrol activity in detached leaves, significantly reducing the symptoms of maize anthracnose.

### 3.4. Inhibitory Effects of Lipopeptide Extract from B. atrophaeus F4 on Pathogenic Fungal Hyphae

**Figure 5:**
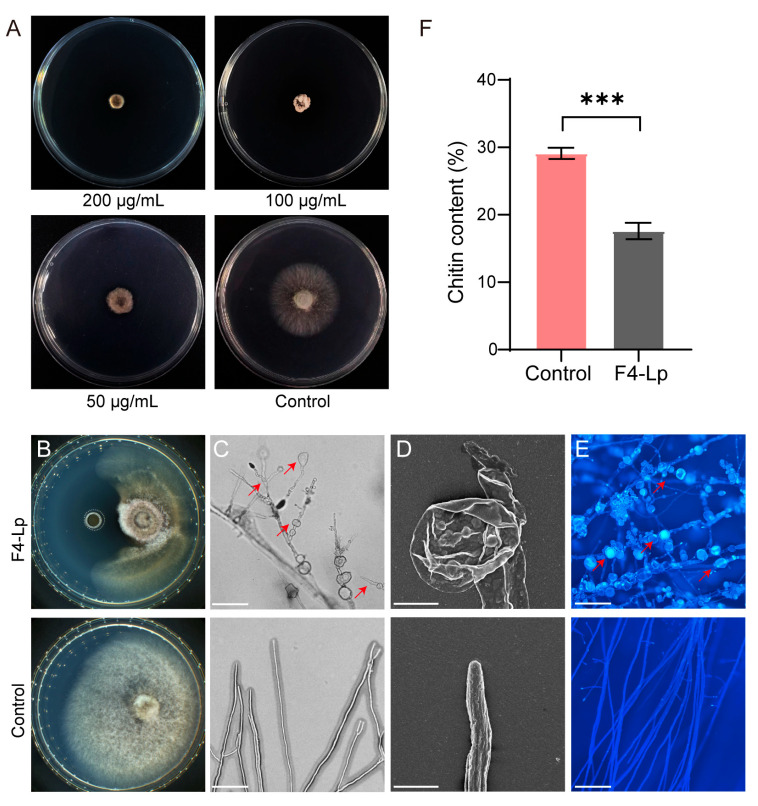
Inhibitory effects of lipopeptide extract from *B. atrophaeus* F4 on the mycelial growth and morphology of the pathogenic fungus. (**A**) Antifungal activity of lipopeptide extract against *C. graminicola* at different concentrations. The control was treated with sterile water. (**B**) Antifungal activity in an agar well diffusion assay using an Oxford cup loaded with 100 µg/mL lipopeptide extract. (**C**) Optical micrographs of mycelia from the inhibition zone margin. The red arrow indicates aberrant morphology. Scale bar, 100 µm. (**D**) Scanning electron microscopy (SEM) images further revealing the ultrastructural damage to the hyphae (bulbous expansion and rupture). Scale bar, 10 µm. (**E**) Fluorescence micrographs of hyphae stained with Calcofluor White (CFW), The red arrow indicates alterations in cell wall integrity. Scale bar, 100 µm. (**F**) Quantitative analysis of chitin content in the mycelial cell walls. ***, *p* < 0.001. For (**B**–**F**), treatment without lipopeptide extract served as control.

To elucidate the biocontrol mechanisms of F4 strain, we examined the inhibitory effects of its lipopeptide extract on hyphal growth of the pathogen *C. graminicola*. Plate inhibition assays revealed that the lipopeptide extract significantly suppressed fungal radial expansion in a concentration-dependent manner. At a concentration of 50 μg/mL, the diameter of the pathogen colonies (2.2 ± 0.3 mm) was significantly reduced compared to the control group (26.4 ± 0.4 mm), with an inhibition rate of 84.5 ± 0.3%. Upon increasing the concentration to 100 μg/mL, the inhibition rate escalated to 99.3 ± 0.4%. At 200 μg/mL, fungal growth was completely inhibited (Figure 5A).

The fungal hyphae in the inhibition zone showed obvious morphological abnormalities, exhibited localized breakage, collapse, distortion, swelling, or shrinkage, with some hyphal tips showing bulbous expansion and rupture, suggesting compromised cell wall integrity. In contrast, the control hyphae treated with sterile water maintained their normal cylindrical structures with smooth surfaces (Figure 5B–D). Calcofluor white (CFW) staining revealed discontinuous or irregularly intensified fluorescence signals in the cell walls of treated hyphae, whereas control hyphae displayed uniform fluorescence distribution (Figure 5E). Quantitative analysis of chitin demonstrated that treatment with 100 μg/mL lipopeptide significantly reduced chitin content to 17.6 ± 1.2 mg/g dry hyphae, compared to 29.1 ± 0.8 mg/g in the control group (*p* < 0.001) (Figure 5F), suggesting an interference with chitin biosynthesis. These findings suggest that the lipopeptide extract from F4 strain effectively inhibits the growth of pathogenic fungal hyphae by disrupting hyphal morphological structure, altering chitin distribution within cell walls, and reducing overall chitin content.

### 3.5. Inhibitory Effects of Lipopeptide Extract from B. atrophaeus F4 on Pathogenic Conidial Germination

**Figure 6:**
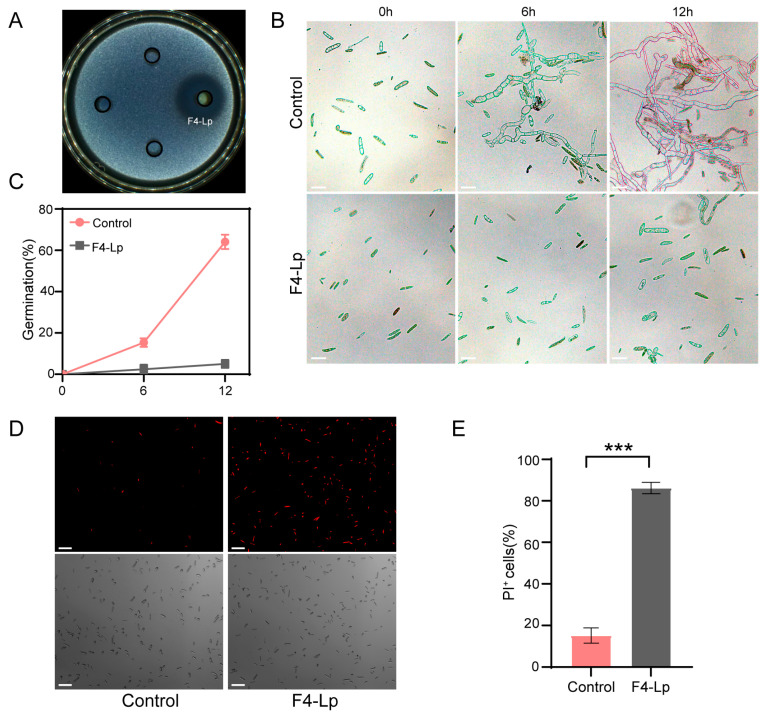
Inhibitory effects of lipopeptide extract *B. atrophaeus* F4 on conidia of the pathogen. Sterile water treatment was used as control, and F4-Lp denotes F4 lipopeptide extract. (**A**) Inhibitory effect of lipopeptide extract on conidia germination. The Oxford Cup on the right is for F4-Lp, while the rest are control. (**B**) Micrographs showing the germination status of conidia at 0, 6, and 12 h. Scale bar, 20 µm. (**C**) Conidial germination rates. (**D**) Fluorescence micrographs of conidia stained with propidium iodide (PI) to assess cell membrane integrity. PI-positive staining (red fluorescence) indicates dead or membrane-compromised conidia. Scale bar, 10 µm. (**E**) Statistical analysis of PI-positive conidia. ***, *p* < 0.001.

We further evaluated the impact of lipopeptide extract from F4 strain on conidia of the pathogen. Agar diffusion assays revealed a distinct inhibition zone with a diameter of 2.5 ± 0.2 cm surrounding the Oxford cup containing 100 μg/mL of lipopeptide extract, whereas no such inhibition zone was observed in the water control group (Figure 6A). The germination rate of control conidia reached 15.3 ± 2.1% at 6 h and 64.1 ± 3.5% at 12 h, accompanied by hyphal growth. In contrast, almost no conidial germination was observed in the group treated with 100 μg/mL lipopeptide extract within the 0–12 h timeframe (Figure 6B,C).

To elucidate the reason underlying the inhibition of conidial germination, we assessed the integrity of the conidial cell membrane. The results indicated that the PI-positive staining rate was 86.2 ± 3.0% in the treated group, significantly higher than the 15.2 ± 4.9% observed in the control, suggesting that the lipopeptide extract disrupts the conidial membrane structure, leading to cytoplasmic leakage (Figure 6D,E).

In conclusion, the lipopeptide extract from F4 strain effectively inhibits conidial germination by compromising membrane integrity and permeability.

### 3.6. Analysis of Lipopeptide Extract Components by MALDI-TOF MS

**Figure 7:**
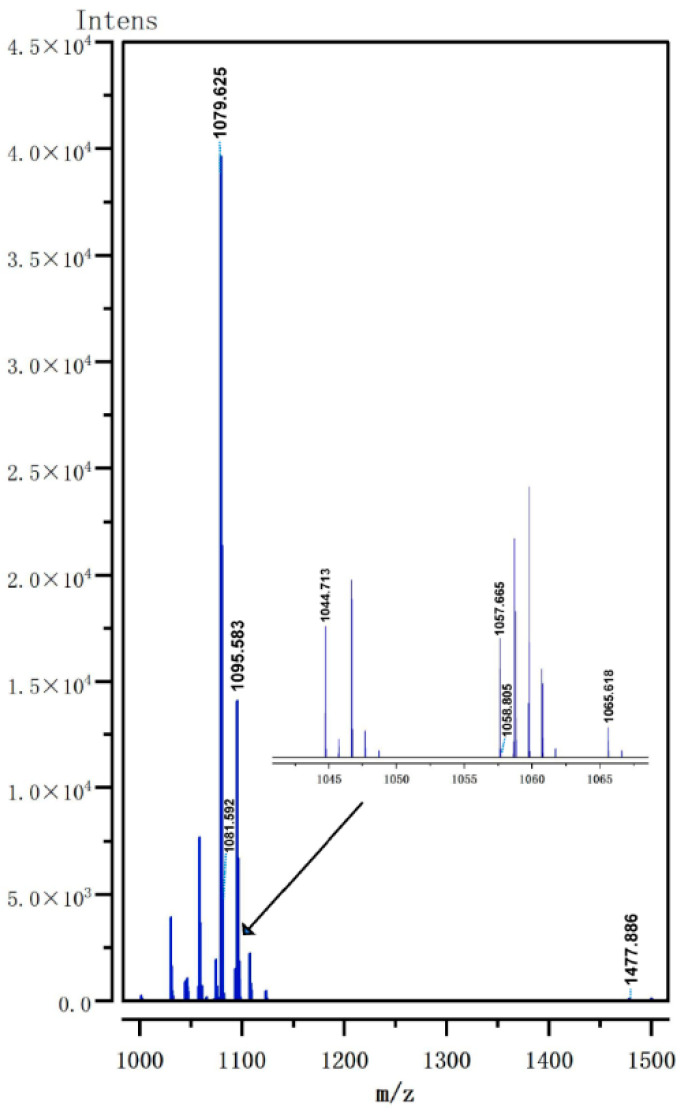
MALDI-TOF mass spectra of the lipopeptide extracts obtained from F4 cell-free fermentation supernatant in the *m*/*z* range from 1000 to 1500.

As shown in the mass spectrum, the lipopeptide extract exhibited eight characteristic peaks consistent with or closely matching previously reported mass values for known lipopeptides, with *m*/*z* of 1044.713, 1057.665, 1058.805, 1065.618, 1079.625, 1081.592, 1095.583, and 1477.886. These peaks were identified as C14 surfactin [M + Na]^+^, C15 iturin [M + H]^+^, C15 surfactin [M + Na]^+^, C14 iturin [M + Na]^+^, C15 iturin [M + Na]^+^, C14 iturin [M + K]^+^, C15 iturin [M + K]^+^, and C15 fengycin B [M + H]^+^, respectively (Figure 7, Table 4). These findings are consistent with the analysis of two lipopeptides, surfactin and fengycin, in our antiSMASH secondary metabolite analysis.

### 3.7. Antifungal Spectrum and Other Biocontrol-Related Characteristics of B. atrophaeus F4

**Figure 8:**
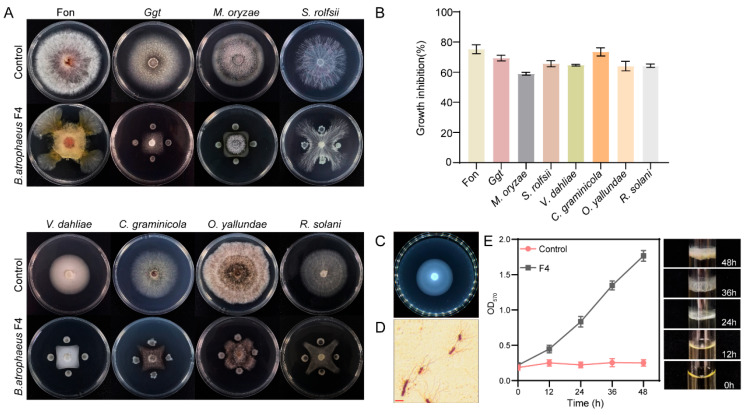
Analysis of antifungal spectrum, motility, and biofilm formation ability of *B. atrophaeus* F4. (**A**) Antagonistic activity against plant pathogenic fungi. (**B**) Inhibition rate against pathogenic fungi. (**C**) Motility tested with swimming media. (**D**) Flagellar morphology under optical microscope. scale bar, 5 µm. (**E**) Biofilm formation ability. (**Right**), images of biofilm development at 0–48 h. (**Left**), Quantitative analysis of biofilm biomass over time. LB medium without bacterial inoculation was used as control.

In addition to exhibiting substantial biocontrol activity against *C. graminicola*, this study further evaluated the in vitro antagonistic effects of F4 strain against other plant pathogenic fungi, as well as its potential biocontrol-related traits. Using the plate confrontation assay, F4 strain exhibited inhibitory effects against all eight tested plant pathogenic fungi, including *C. graminicola*. The most pronounced inhibition was observed against *C. graminicola*, with an inhibition rate of 75.8 ± 0.7% at 5 days. Inhibition rates for *Fusarium oxysporum* f. sp. *niveum* (Fon), *Gaeumannomyces graminis* var*. tritici* (Ggt), *Magnaporthe oryzae*, *Sclerotium rolfsii*, *Verticillium dahlia*, *O. yallundae* and *Rhizoctonia solani* were 75.2 ± 0.9% (5 days), 73.9 ± 0.9% (7 days), 53.0 ± 0.4% (7 days), 67.7 ± 0.3% (5 days), 65.6 ± 0.6% (8 days), 66.3 ± 0.4% (6 days), and 64.9 ± 0.9% (5 days), respectively, indicating the broad-spectrum antifungal activity of F4 strain (Figure 8A,B).

Motility, biofilm formation, and extracellular enzyme secretion are critical traits for the colonization for biocontrol bacteria to colonize and exert their biocontrol effects on the host surface. Semi-solid medium assays revealed that F4 strain formed distinct motility halos, and microscopic observations confirmed the presence of peritrichous flagella (Figure 8C,D).

When cultured statically in LBS liquid medium, F4 strain formed robust, ring-shaped biofilms at the air–liquid–solid interface, which remained intact after gentle agitation. Quantification using crystal violet staining indicated a gradual accumulation of biofilm biomass, reaching an optical density of 1.77 ± 0.08 at 570 nm after 48 h (Figure 8E). At this time point, a continuous, thick, and structurally stable milky-white biofilm with pronounced wrinkling was observed at the air–liquid interface, which could be lifted intact (Figure S1A). Furthermore, F4 strain produced clear hydrolytic zones on skim milk agar and iodine-stained starch plates (Figure S1B,C), confirming its ability to secrete extracellular proteases and amylases.

In conclusion, F4 strain not only exhibits broad-spectrum antifungal activity but also demonstrates well motility, biofilm-forming capacity, and multiple extracellular enzyme-secreting capabilities. These characteristics collectively enhance its potential for colonization and biocontrol efficacy on plant surfaces.

## 4. Discussion

In this study, a bacterial F4 strain with significant control efficacy against maize anthracnose from the Tianshan Mountain soil in Xinjiang, China. Greenhouse trials confirmed that the fermentation broth of F4 markedly reduced the disease index of maize anthracnose, achieving a control efficacy of up to 79.78%. The strain was identified as *B. atrophaeus*. Beyond *C. graminicola*, F4 strain exhibited broad-spectrum antifungal activity against multiple phytopathogenic fungi, with in vitro inhibition rates ranging from 53% to 76%. This significantly expands its potential application value as a broad-spectrum biocontrol agent.

Genome sequencing and bioinformatic analysis revealed that F4 strain harbors numerous biosynthetic gene clusters responsible for the synthesis of antimicrobial secondary metabolites, particularly lipopeptides, suggesting their potential role in its biocontrol mechanisms. Lipopeptide compounds of F4 strain were extracted using acid precipitation, and their biocontrol activity was validated on detached leaves. Microscopic observations revealed that the lipopeptide extract inhibited fungal hyphae growth by disrupting the cell wall integrity of the anthracnose pathogen. Further assays indicated that the lipopeptide extract significantly suppressed chitin synthesis in the pathogen. As chitin is a critical structural component of fungal cell walls, its inhibition severely compromises cell wall strength, which may represent a core mechanism underlying the observed cell wall integrity impairment and mycelial growth inhibition. Moreover, the lipopeptide extract impaired membrane integrity and permeability, leading to the loss of conidial germination capacity in the pathogen, a crucial step in blocking primary infection and disease transmission cycles. MALDI-TOF MS analysis showed the presence of surfactin, iturin, and fengycin B in the lipopeptide extract of strain F4. The identification of these compounds provides evidence for understanding the biocontrol mechanism of F4 strain.

Based on these findings, we propose a mechanistic model for the biocontrol activity of F4 strain: the lipopeptide antimicrobial compounds (surfactin, iturin, and fengycin B) produced by F4 strain inhibit chitin synthesis in *C. graminicola*, disrupt its cell wall structure, and further damage membrane integrity, ultimately leading to mycelial growth arrest and conidial germination failure, thereby achieving effective disease control.

Numerous studies have reported the potent antifungal activity of lipopeptides, demonstrating their significant role in biocontrol against some plant diseases [58,59,60,61]. Their mechanisms of action primarily include: (1) binding to fungal membrane sterols (e.g., ergosterol) to form transmembrane ion channels, causing ion leakage, membrane potential disruption, and cellular content loss. This is a mechanism commonly observed in iturin and fengycin lipopeptides; (2) reducing surface tension to facilitate penetration of other antimicrobial compounds (e.g., surfactin) while inducing hyphal abnormalities (swelling, distortion) and spore germination inhibition [61,62,63,64]. The variation in biocontrol efficacy among different strains may stem from inherent differences in lipopeptide types, congener ratios, and production yields. Additionally, strain-specific regulatory systems such as quorum sensing and environmental signal responses could influence their biocontrol performance. In this research, we specifically identified the lipopeptides surfactin, iturin, and fengycin B from F4 strain as effective agents against maize anthracnose. We elucidated their mechanism of action, which involves the inhibition of chitin synthesis and disruption of cell wall integrity. Chitin, a major component of the cell walls of higher terrestrial fungi, constitutes a critical target for antifungal strategies. Although host plants can induce the secretion of chitinases to degrade pathogen cell walls during infection [15,65], our study concentrated on the direct effects of bacterial metabolites involved in biocontrol, rather than on plant-derived chitinases. Calcofluor white staining of fungal hyphae treated with lipopeptide extract revealed uneven fluorescence distribution, indicating disrupted chitin deposition and cell wall damage. Quantitative analysis confirmed significant reduction in cell wall chitin content. However, due to technical challenges in compound isolation, we could not determine whether individual lipopeptide components act independently or synergistically, which is an important aspect requiring further investigation.

The biocontrol efficacy of *B. atrophaeus* F4 is primarily mediated by lipopeptides, a strategy well-documented in other plant-beneficial *Bacillus* species. Its lipopeptide profile—surfactin, iturin, and fengycin—is highly analogous to the antimicrobial arsenal of *B. subtilis* and *B. velezensis*, which are known for broad-spectrum activity [66,67]. However, strain-specific variations in the relative abundance or congeners of these lipopeptides may account for differences in efficacy and target specificity.

Although this study focuses on *C. graminicola*, the core mechanisms identified—targeting chitin synthesis and membrane integrity—hold promise for managing other *Colletotrichum* pathogens. Species such as *C. gloeosporioides* (fruit anthracnose) and *C. higginsianum* (a crucifer pathogen model) share a conserved hemibiotrophic lifestyle and rely on structurally similar chitin-containing cell walls. Prior studies confirm that bacillomycin and fengycin families from *B. velezensis* are effective against *C. gloeosporioides* via membrane permeabilization [68,69]. Nevertheless, differences in spore morphology and infection biology among *Colletotrichum* species may influence the efficacy of F4’s lipopeptides, warranting further testing to define its full application potential.

The hemibiotrophic fungus *C. graminicola* produces two types of asexual conidia: larger falcate conidia and smaller oval conidia. Both are infectious and cause maize anthracnose [70]. Falcate conidia typically form in necrotic lesions on maize tissues. They efficiently produce appressoria, facilitating penetration of intact leaves, and serve as the primary propagules for field dispersal. In contrast, oval conidia arise in parenchyma cells of infected leaves or stems and spread longitudinally through vascular bundles or stem cortex, playing a key role in intra-host distribution and colonization [71,72]. Notably, falcate conidia secrete the self-inhibitor glutamine, whereas oval conidia lack this compound [37].

To investigate the effects of lipopeptide extract on fungal conidia, we employed the oval conidia described above. These conidia are more easily obtained than falcate conidia, requiring only 7–10 days of cultivation in a liquid medium followed by filtration, whereas falcate conidia necessitate 2–3 weeks of growth on oat meal agar (OMA) solid medium [73]. Treatment with 100 μg/mL lipopeptide extract completely inhibited the germination of oval conidia. Further propidium iodide (PI) staining revealed compromised membrane integrity and permeability in the treated conidia. Although falcate conidia were not tested, these results strongly suggest potent antifungal activity of lipopeptide extract from F4 strain against fungal conidia. Given the absence of secondary field transmission, oval conidia were also used for pot experiments and detached leaf bioassays, where they successfully induced typical anthracnose symptoms on susceptible maize B73 seedlings and leaves, consistent with prior reports [2,20].

An outstanding biocontrol strain typically possesses multiple capabilities, including antibiotic production, siderophore synthesis, extracellular enzyme secretion, and induced systemic resistance (ISR) [74]. These functions are usually not isolated but operate through synergistic mechanisms. Biofilms enhance bacterial adhesion to plant surfaces and stress tolerance, providing a stable microenvironment for antibiotic and enzyme production. Motility facilitates bacteria spread in rhizosphere or phyllosphere, quickly occupying more ecological niches, and ensuring population establishment. Extracellular enzymes degrade pathogen structures, releasing nutrients to support bacterial growth and competition. Certain metabolites such as siderophores and lipopeptides, perform multiple roles such as antimicrobial agents, ISR elicitors and promoting plant growth [75,76]. F4 strain exhibits multiple biocontrol traits, including motility, biofilm formation, and extracellular enzyme production. These phenotype-associated genes are the molecular basis for the strain to exercise biocontrol mechanisms. As expected, multiple functional genes corresponding to the observed phenotypes were identified in the F4 genome. These include biosynthetic gene clusters for antimicrobial compounds such as surfactin (e.g., *srfAA*, *srfAB*, *srfAC*), bacillaene (e.g., *baeC*, *baeD*, *baeG*, *baeJ*, *baeL*, *baeM*, *baeN*), fengycin (e.g., *fenA-E*), and sporulation killing factor (e.g., *skfA-C*), which collectively constitute the genetic basis for the broad-spectrum antifungal activity of F4 strain. Flagella-associated genes (e.g., *motA*, *motB*, *flgB–flgD*, *flgG*, *fliE–fliM*, etc.) were also annotated, encoding proteins essential for flagellar assembly and thus underpinning motility and colonization potential. Furthermore, biofilm-related genes, including those involved in exopolysaccharide synthesis (*eps*), the *tapA-sipW-tasA* operon, and the *sinI*/*sinR* regulatory system, were identified and likely contribute to the robust biofilm formation observed experimentally. Additionally, genes encoding extracellular enzymes such as proteases and α-glucosidase were detected, highlighting their crucial role in nutrient degradation and ecological adaptation.

Although the F4 strain has many advantages related to biocontrol, the specific contribution of each trait to overall biocontrol efficacy remains unclear. The application of CRISPR/Cas9-mediated gene knockout technology, as demonstrated in studies on *C. graminicola*, could be utilized to construct pathogen mutants for precise functional analysis. Moreover, the current findings are predominantly based on in vitro and greenhouse experiments. To assess the practical applicability of F4 strain, subsequent research needs to be conducted in field settings to verify the stability of its control effects. This research should also focus on the influence of complex environmental factors, such as temperature, humidity, and soil microbial communities, on its biocontrol efficacy.

## 5. Conclusions

This study achieved its objective of isolating and characterizing a novel biocontrol agent against maize anthracnose. The key findings are: (1) *B. atrophaeus* F4 was successfully isolated and demonstrated high control efficacy (up to 79.78%) against *C. graminicola* in greenhouse trials, alongside broad-spectrum antifungal activity. (2) Its biocontrol mechanism is primarily mediated by lipopeptides (surfactin, iturin, and fengycin B), which inhibit fungal growth by disrupting chitin synthesis, cell wall integrity, and membrane function. (3) Genomic analysis corroborated these traits by revealing corresponding biosynthetic gene clusters and genes associated with motility and biofilm formation.

In conclusion, *B. atrophaeus* F4 represents a highly promising candidate for the development of an environmentally friendly biocontrol product. To translate this potential into practical application, future research should focus on: (i) validating its efficacy and stability under field conditions; (ii) elucidating the individual and synergistic roles of its lipopeptide components; and (iii) developing effective formulations to enhance its shelf-life and delivery.

## Figures and Tables

**Table 1:** Fungal pathogens used in this study.

Abbreviation	Full Name	Disease Caused
Fon	*Fusarium oxysporum* f. sp*. niveum*	watermelon Fusarium wilt [30]
*Ggt*	*Gaeumannomyces graminis* var. *tritici*	wheat take-all [31]
*Monzae*	*Magnaporthe oryzae*	rice blast [32]
*S. rolfsii*	*Sclerotium rolfsii*	peanut stem rot [33]
*V. dahliae*	*Verticillium dahliae*	verticillium wilt [34]
*C. graminicola*	*Colletotrichum graminicola*	maize anthracnose [20]
*O. vallundae*	*Oculimacula vallindae*	sharp eyespot [35]
*R. solani*	*Rhizoctonia solani*	seedling blight [36]

**Table 2:** Biocontrol efficacy of *B. atrophaeus* F4 against maize anthracnose.

Treatment	Disease Index	Disease Incidence	Control Efficacy
LB medium (negative control)	0.00	0.00	_
*C. graminicola* (positive control)	0.89	95.00%	_
*C. graminicola* + F4 Broth	0.18	20.00%	79.78%
*C. graminicola* + F4-S	0.20	22.00%	77.53%

Abbreviations: F4 Broth, F4 fermentation broth; F4-S, F4 cell-free fermentation supernatant.

**Table 3:** The prediction results of secondary metabolite gene clusters of *B. atrophaeus* F4.

Region	Location (bp)	Predicted BGC Type(s)	Most Similar Known Cluster	Similarity ^a^	Evidence Level ^b^
1	205,818–252,822	NRPS, Sactipeptide, Ranthipeptide	Sporulation killing factor	High	Strong
2	374,774–440,187	NRPS	Surfactin	High	Strong
3	1,162,693–1,183,514	Terpene	- ^c^	-	Putative
4	1,777,754–1,893,259	transAT-PKS, T3PKS, NRPS	Bacillaene	High	Strong
5	1,994,803–2,141,863	NRPS, Betalactone, transAT-PKS	Fengycin	High	Strong
6	2,153,077–2,174,966	Terpene	-	-	Putative
7	2,246,856–2,286,953	T3PKS	1-Carbapen-2-em-3-carboxylic acid	low	Weak/Putative
8	3,139,212–3,191,077	NRP-Metallophore, NRPS	Bacillibactin	High	Strong
9	3,204,298–3,234,401	Azole-containing RIPP	-	-	Putative
10	3,693,340–3,714,949	Sactipeptide	Subtilosin A	High	Strong
11	3,952,188–3,973,886	Epipeptide	-	-	Putative

^a^. Similarity, As reported by antiSMASH, indicating the sequence similarity to the MIBiG database reference cluster. ^b^. Evidence Level, Strong indicates a high-confidence prediction with significant similarity to a well-characterized known cluster. Putative indicates a region encoding the necessary biosynthetic machinery but with low or no significant similarity to known clusters, suggesting potential novelty. Weak indicates a low-similarity match, requiring further validation. ^c^. Dash (-) indicates that no significant similarity to a known cluster was found. Abbreviations: BGC, Biosynthetic Gene Cluster; NRPS, Nonribosomal Peptide Synthetase; PKS, Polyketide Synthase; transAT-PKS, trans-Acyltransferase PKS; T3PKS, Type III PKS; RIPP, Ribosomally synthesized and Post-translationally modified Peptide.

**Table 4:** The lipopeptides presence in cell-free fermentation supernatant extract of *B. atrophaeus* F4 by MALDI-TOF MS.

No.	Mass Peak (*m*/*z*)	Family	Assignment	References
1	1044.713	C14 surfactin	[M + Na]^+^	[54]
2	1057.665	C15 iturin	[M + H]^+^	[55]
3	1058.805	C15 surfactin	[M + Na]^+^	[55]
4	1065.618	C14 iturin	[M + Na]^+^	[56]
5	1079.625	C15 iturin	[M + Na]^+^	[55]
6	1081.592	C14 iturin	[M + K]^+^	[54]
7	1095.583	C15 iturin	[M + K]^+^	[54]
8	1477.886	C15 fengycin B	[M + H]^+^	[57]

## Data Availability

The original contributions presented in this study are included in the article and Supplementary Materials. Further inquiries can be directed to the corresponding authors.

## Supplementary Materials

The following supporting information can be downloaded at: https://www.mdpi.com/article/10.3390/microorganisms14010047/s1, Figure S1. Analysis of biofilm formation and extracellular hydrolase production by *Bacillus atrophaeus* F4; (A) Biofilm formation at the air–liquid interface. (B) Detection of protease production on a skim milk agar plate. The transparent zone around the colony indicates casein degradation. (C) Detection of amylase production on a starch agar plate. The clear zone visible after iodine flooding demonstrates starch hydrolysis.
