# Supplementary material for: Screening and Action Mechanism of Biological Control Strain Bacillus atrophaeus F4 Against Maize Anthracnose
Source: Microorganisms. 2025 Dec 25;14(1):47. doi: 10.3390/microorganisms14010047 (PMC12844051; doi:10.3390/microorganisms14010047)
Supplement: Supplementary file 1 [file microorganisms-14-00047-s001.zip › microorganisms-4041631-supplementary.pdf]

Figure S1

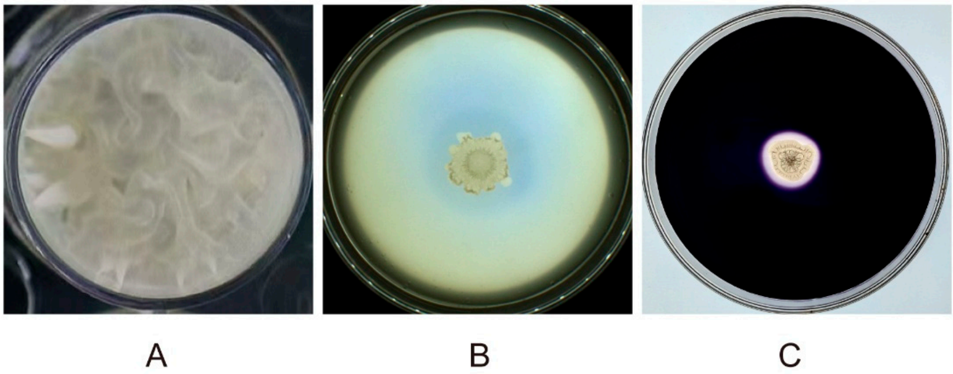

Figure S1. Analysis of biofilm formation and extracellular hydrolase production by *Bacillus atrophaeus* F4.

(A) Biofilm formation at the air-liquid interface.

(B) Detection of protease production on a skim milk agar plate. The transparent zone around the colony indicates casein degradation.

(C) Detection of amylase production on a starch agar plate. The clear zone visible after iodine flooding demonstrates starch hydrolysis.
